# How News Agencies’ Twitter Posts on COVID-19 Vaccines Attract Audiences’ Twitter Engagement: A Content Analysis

**DOI:** 10.3390/ijerph19052716

**Published:** 2022-02-25

**Authors:** Di Wang, Jiahui Lu

**Affiliations:** 1 Faculty of Humanities and Arts, Macau University of Science and Technology, R322, Avenida Wai Long, Taipa, Macao 999078, China; dwang@must.edu.mo; 2 School of New Media and Communication, Tianjin University, No. 92, Weijin Road, Nankai District, Tianjin 300072, China

**Keywords:** COVID-19, COVID-19 vaccine, health belief model (HBM), Twitter, content analysis

## Abstract

As the most important global news distributors, the big three international news agencies’ reports about COVID-19 vaccines have a great influence on people’s understanding of them. Based on the health belief model (HBM), we examined which constructs in the HBM were related to audiences’ Twitter engagement and the differences among the agencies. We content-analyzed 1162 COVID-19 vaccine-related tweets from three international news agencies’ Twitter accounts (@AFPespanol, @AP, @Reuters) from 2 December 2020 to 31 January 2021. The results showed that the most-used HBM construct was barriers, followed by benefits, susceptibility, cues to action, severity, and self-efficacy. About half of the tweets used a positive tone and nearly half of the tweets used a neutral tone, while only 3.1% of the tweets used a negative tone. Reuters used a significantly more negative tone, more neutral tone, and less positive tone than was expected. AFP used a significantly more positive tone and less neutral tone than was expected. The effectiveness of utilizing HBM constructs for vaccination promotion strongly depends on the audience context. The use of HBM constructs for vaccination was generally effective for Reuters but seems to have backfired for AFP.

## 1. Introduction

Since its appearance in December 2019, coronavirus disease (COVID-19) has become a global pandemic, and to date, it has not been effectively curbed. In particular, the emergence of COVID-19 variants such as Delta and Omicron led to a new wave of COVID-19 in many countries. Although countries around the world implemented a series of public health and social measures, such as movement control, social distancing, and personal measures [1], people still urgently need an effective vaccine to control the pandemic. The research and development of COVID-19 vaccines have recently been the focus of worldwide attention. Although Russia approved its COVID-19 vaccine for the market as early as 11 August 2020, large-scale vaccine approval began in December 2020. As of December 2020, the UK (2 December) [2], the US (11 December) [3], the EU (21 December) [4], China (31 December) [5], and other countries and regions successively approved the use of their own COVID-19 vaccines.

Previous research showed that taking a COVID-19 vaccine, and taking vaccines in general, is a controversial and debatable topic in many societies. Some people doubt the effectiveness of the vaccines, while others worry about possible side effects [6]. Cornwall [7] pointed out that a series of reports on side effects on social media increased the public’s hesitation regarding COVID-19 vaccines and distrust of the vaccination plan. Lazarus and colleagues’ research found that, although COVID-19 vaccines are proven to be safe and effective, only 71.5% of the respondents from 19 countries or regions had the intention of vaccination [8].

Reducing public vaccine hesitation and promoting vaccination is an important task in the fight against COVID-19 [9]. In terms of the discursive construction of the reality, news media could play an important role [10]. As the most important global news distributors, international news agencies’ reports about COVID-19 vaccines have a great influence on people’s understanding of vaccines. Research found that international news agencies can set not only the agenda of print news media but also the agenda of online news media [11]. Among the many international news agencies, the big three agencies, namely, the Associated Press (AP), Reuters (Reuter Ltd.), and Agence France-Presse (AFP), have continuously provided non-partisan news to all subscribers [12]. In recent years, these agencies have also used Twitter to communicate with their audiences. However, researchers pointed out that Twitter is often used as a one-way information channel, and senders often do not pay enough attention to the needs and preferences of the public [13,14]. To make full use of the advantages of Twitter, scholars argued that senders should improve their social media content to stimulate audience engagement [15]. Audience engagement can improve the awareness of health-related information, a sense of belonging, and social connection [16].

To measure Twitter engagement, appropriate evaluation criteria and metrics need to be applied. Many scholars used specific behavior indicators, such as the number of “likes” and “retweets”, to measure Twitter engagement [17,18]. A “like” is a way for users to indicate their interest in the tweet [19]. When users retweet a post, it indicates that, after processing the information, they consciously decided to share it [20] and hoped to spread the information that they believe has news value [21]. By using likes and retweets, followers show more attention, and their level of participation increases [22].

In this study, we aimed to explore how news agencies’ Twitter posts on COVID-19 vaccines attracted audiences’ Twitter engagement. By inducing positive Twitter engagement, news agencies could potentially change their audiences’ views on COVID-19 vaccines and further increase the inoculation rate. To this end, we used a content analysis to examine the information that was posted about COVID-19 by three news agencies’ Twitter accounts. Specifically, we examined which constructs in the health belief model (HBM) were related to audiences’ Twitter engagement and whether there were differences among the three news agencies. While traditional research on health information communication using content analysis can only study the health information itself, the current study was designed to take advantage of social media and to test the communication effect of different health messages. The results of the study can also provide references for health communication design on social media in the future.

### 1.1. Health Belief Model Constructs and Twitter Engagement

The health belief model (HBM) is a theoretical model based on psychology and sociology [23], emphasizing that individuals’ adoption of health behavior is affected by a series of beliefs, including (a) whether they are vulnerable to the disease or health risks (perceived susceptibility), (b) the severity of the disease (perceived severity), (c) the difficulty of taking preventative actions (perceived barriers), (d) the benefits of taking those actions (perceived benefits), (e) whether they can successfully implement the recommended health behavior (self-efficacy), and (f) whether they are prepared to adhere to appropriate health measures [24]. Although HBM was originally proposed and examined as a psychological model to predict people’s health behavior, such as with vaccine uptake [25], it has been used to guide the information design of various health intervention plans and activities [26]. Recently, researchers have begun to study the expression of HBM concepts on various media platforms, including Twitter [16,27,28,29], Facebook [30,31], and Pinterest [32]. Understanding the frequency of the HBM constructs appearing on the three news agencies’ Twitter feeds and the extent to which these constructs can promote Twitter engagement will help media practitioners and health professionals determine publicity strategies conducive to good health attitudes and behaviors.

### 1.2. News Agencies, HBM Constructs and Twitter Engagement

News agencies were jointly initiated by newspapers in the 1830s and 1840s. Their initial purpose was to reduce production costs and expand the scope of foreign correspondence [33,34]. With the decline of the American agency UPI in the late 1990s, scholars argued that three large news agencies (i.e., the Associated Press, Reuters and Agence France-Presse) dominate the worldwide flow of today’s news [35]. The Associated Press (AP) is a non-profit news agency founded in New York City in 1846. Data from 2020 showed that it operates in 245 locations in 97 countries [36]. Reuters is an international news agency founded in London in 1851. In 2008, it was acquired by the Thomson Corporation and became the media division of Thomson Reuters. Reuters has received government subsidies in the past [37]. It became a listed company in 1984 [38] and operates in over 100 countries [39]. Agence France-Presse (AFP) is an international news agency founded in Paris in 1835 as Havas. It was renamed Agence France-Presse in 1944. Founded as a state enterprise, AFP still receives subsidies from the French government [40]. AFP’s staff members in 2021 were located in more than 260 locations in 151 countries [41]. The big three news agencies enjoy a good reputation for providing accurate, fast, and unbiased reports [33]. Although international news agencies have long been the core of global news distributors, they have not attracted much academic interest. Because international news agencies have created an objective and fact-oriented ideal public image [42], their professionalism is considered indisputable [43]. The few studies that have examined the news agencies’ objectivity have confirmed such an image. For example, Horvit [44] compared six news agencies’ reports of the 2003 US–Iraq conflict and found that AP, Reuters and AFP were more balanced in their reporting than Xinhua news agency, Information Telegraph Agency of Russia (ITAR-TASS) and Inter Press Service (IPS) were.

Scholars believe that the three major news organizations monopolize or at least dominate the global news flow, and thus play significant roles in people’s understanding of global issues [33,35]. Wu [45] found that developing countries rely primarily on the big three news agencies for international news. That is, the big three news agencies often serve as agenda setters for the local media and citizens of those countries, not only regarding “what to think about” (first level agenda setting) but also “how to think about” a global issue (second level agenda-setting) [46].

Although the three news agencies share the same principle of objectivity, their relationship is both competitive and cooperative [43]. Today, all three news agencies post news tweets through their Twitter accounts to reach global audiences. At the time of our study, the number of followers was 15,077,382 for AP, 1,979,830 for AFP, and 23,204,432 for Reuters. Given their vital role as worldwide purveyors of information, how these news agencies use Twitter to report on a significant issue such as COVID-19 vaccines takes on added importance. Using the HBM, we aimed to explore the differences among the three news agencies in terms of the HBM constructs used and their different impact on Twitter engagement variables. Therefore, we posed the following questions:

RQ1: Are there differences among the three news agencies in applying the HBM concepts (susceptibility, severity, benefits, barriers, cues to action and self-efficacy) when using Twitter to report on COVID-19 vaccines?

RQ2: Are there differences among the three news agencies in the impact of the HBM concepts (susceptibility, severity, benefits, barriers, cues to action and self-efficacy) on Twitter engagement?

### 1.3. Sentiment with Regard to COVID-19 Vaccines

The vaccine controversy began in the 1990s when several papers and published books linked vaccines with autism, AIDs and Gulf War syndrome [47]. Although the papers were later discredited and withdrawn, the public still has concerns about the safety of vaccines in general. In terms of the vaccine debate, previous research has shown that social media such as Twitter could set the agenda for other online news media [48].

In terms of COVID-19 vaccines, most studies have found that positive sentiment outnumbered negative sentiment toward COVID-19 vaccines on Twitter [49,50,51]. In contrast, other scholars found an 80% increase in vaccine opposition on Twitter when comparing vaccine opposition four months before and four months after the community spread of COVID-19 in the US [52].

Although big data has been used to show the general pattern of the Twitter sentiment toward COVID-19 vaccines, it is unclear if the same attitude has prevailed in regard to the three news agencies’ tweets. In addition, Yousefinaghani and colleagues [51] found that tweets that were positive toward COVID-19 vaccines motivated higher engagement than other tweets. We aimed to explore whether this is also the case for the three news agencies’ tweets. Therefore, we ask the next research questions:

RQ3: What sentiments were used on Twitter by the three news agencies toward COVID-19 vaccination?

RQ4: Do the sentiments with regarding to COVID-19 vaccines have an impact on Twitter engagement?

## 2. Materials and Methods

### 2.1. Sample

This study used content analysis to examine tweets related to COVID-19 vaccines from three international news agencies’ Twitter accounts (Agence France-Presse (@AFPespanol), Associated Press (@AP), and Reuters (@Reuters)) in the early stages of vaccine approval: from 2 December 2020 (the day the UK approved its first vaccine) to 31 January 2021. This period was chosen for investigation because it is a critical period for the media’s framing of COVID-19 vaccines. For this new kind of vaccine, COVID-19 vaccines, how the media defined it in its first presence could have a primary effect on the public’s view of COVID-19 vaccines.

We used Python 3.8 to retrieve tweets about COVID-19 vaccines on Twitter using Twitter’s Application Programming Interfaces in March 2021. Tweets that contain “COVID/COVID19/COVID-19/COVID2019/coronavirus/corona virus” and “vaccine/vaccines/vaccination/vaccinations/vaccinating/vaccinated” were selected. The keyword searches yielded a total of 3156 tweets, including 180 tweets from the Associated Press, 505 tweets from AFP, and 2471 tweets from Reuters. Due to the large number of articles in Reuters, every fourth article was selected from a randomly selected point to achieve a similar sample size for each news agency. After eliminating unrelated tweets, the final sample consisted of 139 tweets from the Associated Press, 449 tweets from AFP, and 574 tweets from Reuters. In total, 1162 tweets were examined.

The full text and images of each file was examined for coding. All the files were downloaded in English. The data for Twitter-specific variables were downloaded from the Twitter website, including the number of likes and the number of retweets.

Each tweet was examined for one or more of the health belief model constructs (susceptibility, severity, benefits, barriers, cues to action and self-efficacy). Because HBM was originally proposed to measure psychological variables, we adjusted the operational definition of the HBM construct according to the operational definition of Glanz [53] to create variables that could measure media content. For the operationalization of the HBM constructs, see Table 1. Coders first identified whether themes within each construct were present. If any of the themes within a construct was present, we count that the construct variable was present in the article. To further explore the benefit construct, we also coded the sub-themes within the 4.1 and 4.2 theme; see Table 2. We coded the sentiment toward COVID-19 vaccines in general for each tweet (positive, negative, or neutral). All the items were coded as 1 when they were mentioned and 0 when they were not mentioned. Two graduate students who are fluent in English coded all of the files. We calculated inter-coder reliability of the two coders by double-coding a random subsample (n = 281 or 22.2%) of the data. Krippendorf’s alpha ranged from 0.80 to 1.0 for the 36 theme and sub-theme items.

### 2.2. Statistical Procedures

To answer RQ1, we used Chi-square analysis to compare the use of each HBM construct by news agencies. To answer RQ2, we ran a series of nonparametric Mann–Whitney U tests to examine the relationship between the presence of HBM constructs and Twitter engagement variables. To answer RQ 3, we used Chi-square analysis to compare the sentiment on COVID-19 vaccines between the three news agencies. To answer RQ 4, we ran a series of Kruskal–Wallis H tests, nonparametric equivalent of one-way ANOVA, to examine the sentiments of tweets about COVID-19 vaccines’ impact on Twitter engagement.

## 3. Result

### 3.1. Descriptive Statistics

The descriptive statistics are shown in Table 3. Among all 1162 tweets, the most used HBM construct was barriers (n = 684, 58.9%), followed by benefits (n = 359, 30.9%), susceptibility (n = 325, 28%), cues to action (n = 248, 21.3%), severity (n = 231, 19.9%), and self-efficacy (n = 25, 2.2%).

Of the sub-themes of susceptibility, 21.1% (n = 245) mentioned the susceptibility of vulnerable people such as older adults and medical staff to COVID-19, while 11.1% (n = 129) mentioned the susceptibility of the general public to COVID-19. Of the sub-themes of severity, 19.3% (n = 224) mentioned the severity of COVID-19 for the general public, while 1.0% (n = 12) mentioned the severity of COVID-19 for the vulnerable people.

Of the sub-themes of benefits, 30.9% (n = 359) mentioned that vaccines are effective at preventing COVID-19 for individuals, 0.9% (n = 11) mentioned that some vaccines are not effective at preventing COVID-19 for individuals, 1.7% (n = 20) mentioned the benefits of vaccination for society, and 0.1% (n = 1) mentioned that vaccines are not effective at preventing COVID-19 in society.

The most often-mentioned effective COVID-19 vaccines were American vaccines (n = 206; 17.7%), followed by British vaccines (n = 69, 5.9%), Chinese vaccines (n = 33; 2.8%), Indian vaccines (n = 15; 1.3%), and Russian vaccines (n = 14; 1.2%). The most often-mentioned ineffective COVID-19 vaccines were Chinese vaccines (n = 7, 0.6%), American vaccines (n = 6, 0.5%), and Russian vaccines (n = 2, 0.2%).

The most mentioned barriers are access barriers (n = 184, 15.8%), followed by harm barriers (n = 62, 5.3%), and belief barriers (n = 17, 1.5%). The most mentioned cues to action were testimony of ordinary people (n = 114, 9.8%), followed by testimony of celebrities (n = 101, 8.7%), expert recommendations (n = 25, 2.2%) and government recommendations (n = 14, 1.2%).

Of the sub-themes of sentiment with regard to COVID-19 vaccines, 48.5% (n = 564) of tweets were positive in regard to COVID-19 vaccines, 3.1% (n = 36) were negative in regard to COVID-19 vaccines, and 48.4% (n = 562) did not show an attitude toward COVID-19 vaccines.

### 3.2. HBM Constructs Used by Three News Agencies’ Twitter

To answer RQ1, a series of Chi-square analyses were run to compare the use of each HBM construct by news agencies. The results in Table 4 show that the three news agencies’ Twitter accounts used significantly different frequencies of susceptibility (χ^2^ = 16.57, *p* < 0.001), severity (χ^2^ = 65.49, *p* < 0.001), benefits (χ^2^ = 25.02, *p* < 0.001), barriers (χ^2^ = 26.31, *p* < 0.001), and cues to action (χ^2^ = 24.20, *p* < 0.001).

A post hoc analysis showed that the three news agencies emphasized the HBM constructs differently. AP mentioned significantly more susceptibility (n = 59) than the expected count (n = 38.9), while AFP and Reuters showed no differences between the actual frequency and the expected count. AP mentioned significantly more severity (n = 63) than the expected count (n = 27.6), Reuters mentioned significantly less severity (n = 87) than the expected count (n = 114.1), while AFP showed no differences between the actual frequency and the expected count. AP, AFP and Reuters showed no differences between actual frequency and expected count in terms of self-efficacy. AFP mentioned significantly more benefits (n = 170) than the expected count (n = 138.7), Reuters mentioned significantly less benefits (n = 138) than the expected count (n = 177.3), while AP showed no differences between the actual frequency and the expected count. AP mentioned significantly more barriers (n = 101) than the expected count (n = 81.8), Reuters mentioned significantly less barriers (n = 298) than the expected count (n = 337.9), while AFP showed no differences between the actual frequency and the expected count. AFP mentioned significantly more cues to action (n = 126) than the expected count (n = 95.8), Reuters mentioned significantly less cues to action (n = 89) than the expected count (n = 122.5), while AP showed no differences between the actual frequency and the expected count.

In general, Reuters tended to mention less severity, benefits, and cues to action than expected; AP tended to mention more susceptibility, severity, and barriers than expected; and AFP tended to mention more benefits and cues to action than expected.

### 3.3. Differences in the Twitter Engagement

For the entire sample, the mean number of retweets was 96.03 (*SD* = 45.00) and the mean number of likes was 325.39 (*SD* = 95.00). As neither of the engagement variables, the number of retweets or likes were normally distributed, we also examined the median of the engagement variables (Mdn = 45 for retweets and Mdn = 95 for likes), since the median would be a better measure of central tendency than the mean.

In general, AP generated the highest Twitter engagement among the three news agencies.

**Figure 1:**
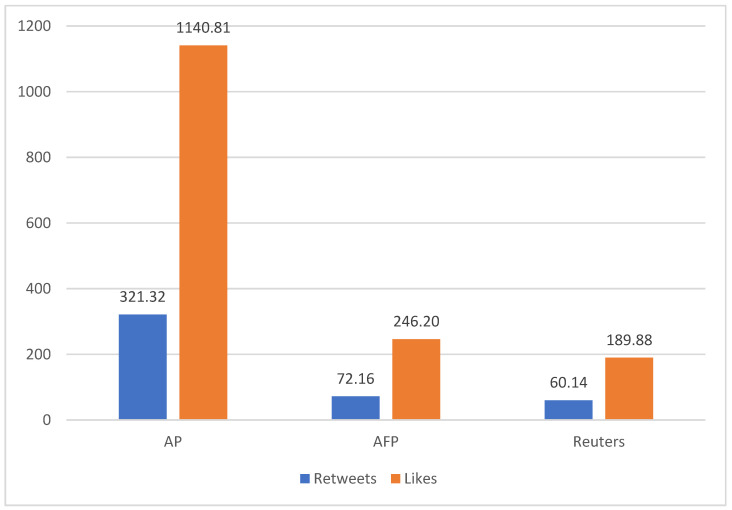
The mean number of retweets and likes in the three news agencies’ Twitter accounts.

AP’s Twitter account generated the highest mean number of retweets (*M* = 321.32, *SD* = 112.00), followed by APF (*M* = 72.16, *SD* = 39.00), and Reuters (*M* = 60.14, *SD* = 38.00). AP’s Twitter account also had the highest mean number of likes (*M* = 1140.81, *SD* = 292.00), followed by Reuters (*M* = 246.20, *SD* = 57.00), and APF (*M* = 189.88, *SD* = 101.00). See Figure 1 for complete results.

**Figure 2:**
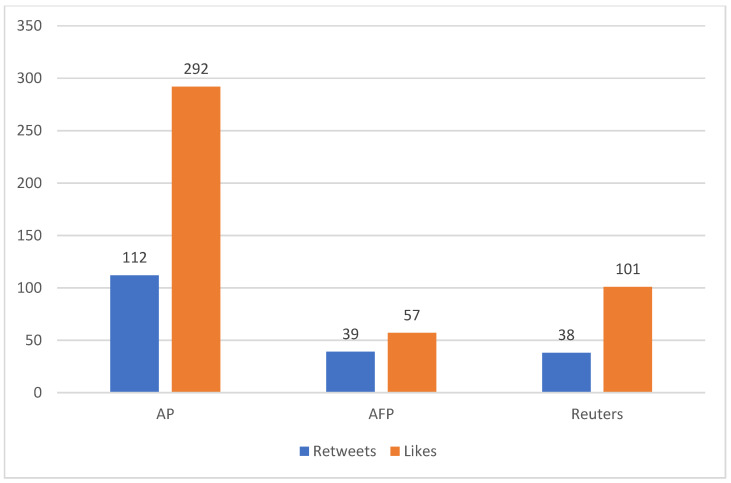
The median number of retweets and likes in the three news agencies’ Twitter accounts.

The median number of retweets was highest for AP’s Twitter account (Mdn = 112), followed by AFP (Mdn = 39), and Reuters (Mdn = 38). The median number of likes was highest for AP’s Twitter account (Mdn = 292), followed by Reuters (Mdn = 101) and AFP (Mdn = 57). See Figure 2 for the complete results.

In general, AP generated the highest Twitter engagement among the three news agencies.

### 3.4. The Effect of HBM Constructs on Twitter Engagement

To answer RQ2, a nonparametric Mann–Whitney U test was used to examine the relationship between the presence of HBM constructs and Twitter engagement variables (likes and retweets), as the Twitter engagement variables were not normally distributed.

For each HBM variable, we first compared the shape of the distribution of likes and retweets of the group with the HBM variable present and the group with the HBM variable present. The results showed that the shapes of the distribution of likes and retweets were different for each of the present and absent two groups. As our two distributions have different shapes, we can only use the Mann–Whitney U test to compare mean ranks rather than medians (Laerd Statistics, 2021).

As can be seen in Table 5, Mann–Whitney U tests showed that, for AP, only one HBM construct (cues to action) can significantly predict Twitter engagement. Tweets emphasizing cues to action were liked more often (mean ranks = 83.39) than tweets that did not (mean ranks = 65.83), Mann–Whitney U = 1307.00, *p* = 0.03.

For AFP, tweets emphasizing three variables (susceptibility, severity, and cues to action) were retweeted less often than those that did not emphasize these variables; tweets emphasizing susceptibility and severity were liked less often than tweets that did not emphasize the two constructs. In contrast, tweets emphasizing self-efficacy were liked and retweeted more often than tweets that did not emphasize self-efficacy.

For Reuters, tweets emphasizing five variables (susceptibility, severity, benefits, barriers, and cues to action) were liked more often than those that did not emphasize those variables. Tweets emphasizing four variables (severity, benefits, barriers, and cues to action) were retweeted more often than those that did not emphasize those variables. In contrast, tweets emphasizing self-efficacy were liked less often (mean ranks = 155.83) than tweets that did not emphasize self-efficacy (mean ranks = 289.60), Mann–Whitney U = 1357.50, *p* = 0.01.

In general, the HBM variables were effective for inducing Twitter engagement for Reuters but demonstrated reversed effects for AFP.

We further examined the effect of sub-themes on Twitter engagement as shown in Table 6. For AP, tweets emphasizing the American vaccines are effective were liked more often than those that did not emphasize this sub-theme. By contrast, tweets emphasizing two sub-themes (Chinese vaccines are effective and access barriers) were liked less often than those that did not emphasize those sub-themes. Tweets emphasizing that Chinese vaccines are effective were retweeted less often than those that did not emphasize this sub-theme.

For AFP, tweets emphasizing six sub-themes (susceptibility of the general public, susceptibility of vulnerable people, severity of the general public, the benefits of vaccination to society, harm barriers and access barriers) were liked less often than those that did not emphasize those sub-themes. Tweets that emphasize six sub-themes (susceptibility of the general public, susceptibility of vulnerable people, severity of the general public, the benefits of vaccination to society, harm barriers, access barriers, testimony of ordinary people) were retweeted less often than those that did not emphasize this sub-theme.

For Reuters, tweets emphasizing seven sub-themes (susceptibility of the general public, susceptibility of vulnerable people, severity of the general public, vaccines are effective at preventing COVID-19 for individuals, American vaccines are effective, American vaccines are NOT effective, and the testimony of celebrities) were liked more often than those that did not emphasize those sub-themes. Tweets emphasizing nine sub-themes (susceptibility of the general public, severity of the general public, vaccines are effective at preventing COVID-19 for individuals, American vaccines are effective, vaccines are NOT effective at preventing COVID-19 for individuals, American vaccines are NOT effective, harm barriers, and the testimony of celebrities) were retweeted more often than those that did not emphasize this sub-theme.

In contrast, tweets emphasizing the benefits of vaccination to society and access barriers were liked and retweeted less often than those that did not emphasize these sub-themes.

Similar to the HBM constructs, in general, the subthemes were effective for inducing Twitter engagement for Reuters, but reducing Twitter engagement for AFP.

### 3.5. News Agencies’ Sentiment Regarding COVID-19 Vaccines

The answer to RQ3 is shown in Table 7. The results showed that 48.5% (n = 564) of the total sample was positive regarding COVID-19 vaccines, 3.1% (n = 36) of the total sample were negative regarding COVID-19 vaccines, while 48.4% (n = 562) of the sample held a neutral sentiment regarding COVID-19.

There were significant differences among the three news agencies in their positive sentiments regarding COVID-19 vaccines (χ^2^ = 14.35, *p* < 0.001). A post hoc analysis showed that AFP demonstrated significantly more positive sentiments (n = 246) than the expected count (n = 217.9), while Reuters used significantly fewer positive sentiments (n = 247) than the expected count (n = 278.6). There were no differences between actual count and the expected count for AP.

There were significant differences between the three news agencies in their negative sentiment regarding COVID-19 vaccines (χ^2^ = 7.78, *p* = 0.02). A post hoc analysis showed that Reuters used significantly more negative sentiments (n = 26) than the expected count (n = 17.8), while there were no differences between the actual count and the expected count for AP and Reuters.

Finally, there were also significant differences between the three news agencies in expressing a neutral sentiment regarding COVID-19 vaccines (χ^2^ = 8.24, *p* = 0.02). A post hoc analysis showed that AFP used significantly less neutral sentiments (n = 195) than the expected count (n = 217.2), while Reuters used significantly more neutral sentiments (n = 301) than the expected count (n = 277.6). There were no differences between the actual count and the expected count for AP.

In general, Reuters tended to show more negative and neutral sentiments toward COVID-19 vaccines, while AFP tended to show more positive sentiments toward COVID-19 vaccines.

### 3.6. News Agencies’ Sentiments Regarding COVID-19 Vaccines and Twitter Engagement

To answer RQ4, we ran a series of Kruskal–Wallis H tests, nonparametric equivalents of one-way ANOVA. For AP, a Kruskal–Wallis H test showed that there were significant differences between the three sentiments in inducing the number of likes, *H* (2) = 6.64, *p* = 0.04. Pairwise comparisons using Dunn’s test showed that for AP, tweets using a positive sentiment (mean rank = 78.08) were liked more than tweets using a neutral sentiment (mean rank = 60.80), *p* = 0.04. A Kruskal–Wallis H test showed that there were no statistically significant differences among the three sentiments in inducing the number of retweets, *H* (2) = 5.04, *p* = 0.08.

For AFP, Kruskal–Wallis H tests showed that there were no statistically significant differences among the three sentiments in inducing retweets, *H* (2) = 2.72, *p* = 0.26, nor were there significant differences among the three sentiments in inducing likes, *H* (2) = 0.64, *p* = 0.73.

For Reuters, a Kruskal–Wallis H test showed that there were significant differences between the three sentiments in inducing the number of likes, *H* (2) = 8.34, *p* = 0.02. Pairwise comparisons using Dunn’s test showed that tweets by Reuters that showed a positive sentiment (mean rank = 310.50) were liked more than tweets using a neutral sentiment (mean rank = 270.12), *p* = 0.01. However, a Kruskal–Wallis H test showed that there were no statistically significant differences among the three sentiments in inducing the number of retweets, *H* (2) = 5.30, *p* = 0.07.

In general, tweets showing a positive sentiment toward COVID-19 vaccines were liked more than tweets showing a neutral sentiment toward COVID-19 vaccines for both AP and Reuters. For details, see Table 8.

## 4. Discussion

### 4.1. Principal Results

This study set out to explore how the “big three” news agencies—the Associated Press, Reuters, and Agence France-Presse—conveyed vaccine-related health information on Twitter, as well as the impact of using the HBM constructs and sentiments on audiences’ Twitter engagement. Our findings demonstrated how the audience contexts of news agencies affect their presentations of vaccine information on Twitter. Findings also revealed the differences in the impacts of using HBM constructs and sentiments on Twitter engagement by news agencies.

Our findings suggest that the presentation of COVID-19 vaccines by international news agencies is both international- and local-audience-oriented. Overall, news agencies tended to emphasize access barriers to COVID-19 vaccines, particularly national and international efforts that were put forth to overcome the barriers. This may be because such efforts can be easily turned into event-based episodic news, and thus are newsworthy [54]. Frequent news about overcoming vaccine access barriers can potentially help to reassure the global public and help them envision a healthy future in the post-COVID era. However, all three news agencies neglected to report ways to increase self-efficacy. This is most likely because concrete guidance for vaccination requires an explicitly localized context, which may not be newsworthy for international audiences.

The three news agencies also emphasized other HBM constructs of the COVID-19 vaccines differently, partly depending on their local audiences. Specifically, AP tended to emphasize the threats of COVID-19. This reporting strategy may result from the fact that the public in the US viewed COVID-19 as only a mild threat to their health in the early months of the pandemic [55]; therefore, AP needed to emphasize its significance to the public. In contrast, AFP, which is still a French-government-funded international agency, was more likely to report the effectiveness of the vaccines and cues to action than the other constructs. This suggests that AFP encouraged their audiences to accept the vaccine by providing effective evidence and recommendations. One possible reason for the framing strategies by AFP is that the COVID-19 vaccine acceptance rate in France is only 58.89%, much lower than the acceptance rate in the US (75.42%) and the UK (71.48%), according to a COVID-19 vaccine survey of 19 countries from June 16 to June 20, 2020 [8]. Compared to the other two agencies, Reuters generally mentioned the HBM constructs less often, corresponding to the fact that the COVID-19 vaccine acceptance rate in the UK is among the highest in the world [8], weakening the need for media mobilization.

A more interesting result is that the use of the HBM constructs showed different impacts on Twitter engagement for the three news agencies. Increased engagements with Reuters’ tweets that emphasized most of the HBM constructs were observed. This is consistent with expectations from the agenda-setting theory, which states that information supplied by the news media about vaccines can influence the public’s attitudes toward and beliefs about them [56]. However, the use of the self-efficacy constructs suppressed Twitter engagement. This may be because tweets emphasizing ways to increase self-efficacy are likely to be localized and the international audiences do not see it as self-relevant.

In contrast, for AFP, tweets emphasizing HBM constructs, including disease threats, barriers, and cues to action generally led to lower Twitter engagements. There may be two explanations for these findings. First, these findings are consistent with those of Guidry et al. [57] regrading Instagram: that posts emphasizing Zika threat received a low Instagram engagement. They explained that this may be because there were no sufficiently high-efficacy responses, such as a vaccine, to prevent Zika disease at that time. Accordingly, people may feel that engagement with such posts is useless. Similarly, audiences of AFP may display maladaptive responses to the heightened COVID-19 threat in the news, as a large proportion of them do not consider vaccination an effective way to prevent the disease. Second, people who do not accept the COVID-19 vaccines may also consider behavioral cues to vaccinate as attempts to manipulate the public, and thereby are resistant to news messages and suppress engagement. This is somewhat consistent with the findings of Ashwell and Murray [58] in Australia and New Zealand between 2016 and 2017. They found that, while news reports about vaccination were predominantly emphasizing its effectiveness in preventing diseases, the vaccination rate in New Zealand was decreasing. They interpreted the findings in terms of emphasis framing theory, which posits that positive news media messages about vaccination can be viewed as advertising and attempting to manipulate the public, leading to public resistance.

The use of the HBM constructs by AP generally did not affect users’ engagement. However, several specific themes did. Interestingly, users resisted engaging in AP tweets that emphasized the effectiveness of the Chinese vaccines, while they liked those that highlighted the effectiveness of the American vaccines. This is likely due to the audiences of AP being largely composed of the US public who perceive high China–US tensions in the political economy [59]. In addition, audiences of AP also engaged more in tweets that provided behavioral cues to vaccinate. This suggests that audiences of AP may be inclined toward COVID-19 vaccination.

Regarding sentiments toward COVID-19 vaccines, international agencies expressed a more positive and neutral sentiment than a negative one. For AP and Reuters, tweets that were positive toward COVID-19 vaccines were liked more than tweets that were neutral toward COVID-19 vaccines. These findings are consistent with Yousefinaghani and colleagues’ study [51], which found that pro-COVID-19 vaccine tweets lead to higher Twitter engagement. However, audiences of AFP did not show more interest in pro-vaccine tweets than the neutral tweets, which corresponded to their low acceptance rate of COVID-19 vaccines [8].

With the above, this study contributes to the research on intentional news agencies by illustrating their difficulties when trying to convey COVID-19 vaccine information to both local and international audiences. As shown above, the effectiveness of utilizing the HBM constructs and positive sentiments on vaccination strongly depends on the audience context. The use of the HBM constructs for vaccination seems to backfire when used on audiences who resist vaccination, as behavioral framing may be viewed as manipulation and threat cues may induce maladaptive responses. In addition, while the use of self-efficacy may be effective in promoting behavioral readiness for vaccination, it may attract only local audiences.

In addition, this study extends the research on the health belief model in the area of message framing and design. News media and health practitioners should employ an audience-based approach, tailoring health messages to specific audiences, when utilizing HBM constructs for persuasion.

### 4.2. Limitations

This study has some limitations that could be addressed by future studies. This study only sampled two months of data from the three news agencies’ Twitter accounts since the COVID-19 vaccine rollout. Future research could examine the change in discourse over time. In addition, we investigated audiences’ Twitter engagement in terms of the number of likes and retweets. Future research could qualitatively analyze Twitter’s comments, which could deepen our understanding of the audiences’ responses to COVID-19 vaccine news.

### 4.3. Conclusions

In conclusion, this research revealed that the “big three” news agencies, including the Associated Press, Reuters, and Agence France-Presse, reported on COVID-19 vaccines differently by highlighting different HBM constructs and sentiments. In addition, the techniques of utilizing HBM constructs and positive sentiments in reporting COVID-19 vaccine tweets attracted audiences in different ways. Audience contexts regarding geographical differences (i.e., local vs. international) and normative attitudes toward COVID-19 vaccines could potentially explain the behaviors of news agencies and their impacts. Our research highlights the potential role of international news agencies in promoting global vaccine coverage, while at the same time revealing their difficulties to feed both local and international audiences.

## Figures and Tables

**Table 1:** Operational definitions and examples.

HBM Constructs	Operational Definition	Themes	Examples
HBM Constructs>Susceptibility	Define population(s) at risk, risk levels; personalize risk based on a person’s features or behavior; heighten perceived susceptibility if too low.	1.1 Susceptibility of the general public	Since the beginning of the pandemic, Latin America has become a hotspot for the virus. Mexico has reported 1,350,079 confirmed cases, Chile has 594,152 confirmed cases, and Costa Rica has 162,990 confirmed cases.
		1.2 Susceptibility of vulnerable people (e.g., older adults, medical staff)	In California, where health care workers will be among the first to be vaccinated, state health officials are prioritizing hospitals that have adequate storage capacity, serve high-risk populations and have the ability to vaccinate people quickly.
Severity	Specify consequences of the risk and the condition.	2.1. Severity of the general public	The scourge has claimed more than 312,000 U.S. lives and killed 1.7 million people worldwide. New cases in the U.S. are running at over 216,000 per day on average. Deaths per day have hit all-time highs, eclipsing 3600 on Wednesday.
		2.2. Severity of vulnerable people (e.g., older adults, medical staff)	Public health data show that nationwide, more than 17,000 COVID-related deaths occurred in nursing homes, and 93% of all deaths from COVID-19 were over 65 years of age.
Self-efficacy	Provide training, guidance in performing action.		How quickly do I need a second COVID-19 vaccine shot? The first COVID-19 vaccines in the U.S. require two doses a few weeks apart.
Benefits	Specify the efficacy of the advised action to reduce risk or seriousness of impact.	4.1. Vaccines are effective at preventing COVID-19 for individuals	It added that the vaccine’s efficacy in preventing COVID-19 was 95 percent, worked uniformly across age groups, genders, and racial groups, as well as people with underlying conditions who are at high risk.
		4.2. Vaccines are NOT effective at preventing COVID-19 for individuals	However, they were significantly less effective at preventing COVID-19 in trial participants in South Africa, where the potent new variant is widespread, compared with countries in which this mutation is still rare, according to preliminary data released by the companies.
		4.3. The benefits of vaccination to society	German travel giant TUI on Thursday posted an annual loss of more than three billion euros as the pandemic devastated tourism, but the group said it was optimistic vaccines would boost travel demand in 2021.
Barriers	Specify the tangible and psychological costs of the advised action.	5.1. Belief barriers (do not catch COVID-19 easily; have a strong immune system.)	He said he was not in a hurry to receive the vaccination. Estefs said: “I have been at home for a year and a half, and I can stay for another two or three months without any problems.”
		5.2. Harm barriers (COVID-19 vaccines can cause more harm than good; Side effects of the vaccine are serious)	“There is not enough research. It is too much too soon. Women are smarter. Men are going to just jump on whatever, they just don’t think. Women are more careful. We are thinking about the future, about side effects about not being sure if it’s safe.”
		5.3. Access Barriers (Unavailability of the vaccine in the right place and time; Cost of the vaccine is a set back)	Karen Stachowiak, a first-grade teacher in the Buffalo area, spent almost five hours on the state hot line and website to land an appointment for Wednesday, only to be told it was canceled. The Erie County Health Department said it scratched vaccinations for over 8000 people in the past few days because of inadequate supply.
Cues to Action	Specify the strategies to activate “readiness.”	6.1. Government’s recommendation	The federal government is seeking to blunt such attitudes with a USD 250 million ad campaign set to roll out this week that will eventually target healthcare workers and vulnerable groups. The pitch touts how vaccines will help beat COVID-19 the same way they defeated smallpox, measles and polio.
		6.2. Experts’ recommendation	At the Tribhuvan University Teaching Hospital in Kathmandu, doctors were encouraging hesitant colleagues to get the vaccine.
		6.3. Testimony of celebrities	Like Vice President Mike Pence Friday and President-elect Joe Biden Monday, the highly visible officials received their vaccinations in a live event to inspire public confidence in the new coronavirus vaccines.
		6.4. Testimony of ordinary people	“I feel so privileged to be the first person vaccinated against COVID-19,” said Keenan, who wore a surgical mask and blue.

**Table 2:** Subthemes within the benefits theme.

Themes	Sub-Themes
4.1. Vaccines are effective at preventing COVID-19 for individuals	4.1.1 Chinese vaccines are effective
	4.1.2 American vaccines are effective
	4.1.3 British vaccines are effective
	4.1.4 Russian vaccines are effective
	4.1.5 Indian vaccines are effective
4.2. Vaccines are NOT effective at preventing COVID-19 for individuals	4.2.1 Chinese vaccines are not effective
	4.2.2 American vaccines are not effective
	4.2.3 British vaccines are not effective
	4.2.4 Russian vaccines are not effective
	4.2.5 Indian vaccines are not effective

**Table 3:** Descriptive statistics.

Variables	n (%)
Health belief model constructs	
1. Susceptibility	325 (28)
2. Severity	231 (19.9)
3. Self-efficacy	25 (2.2)
4. Benefits	359 (30.9)
5. Barriers	684 (58.9)
6. Cues to action	248 (21.3)
Sub-themes of susceptibility	
1.1. General public	129 (11.1)
1.2. Vulnerable people	245 (21.1)
Sub-themes of severity	
2.1. General public	224 (19.3)
2.2. Vulnerable people	12 (1.0)
Sub-themes of benefits	
4.1. Vaccines are effective at preventing COVID-19 for individuals	359 (30.9)
4.1.1. Chinese vaccines are effective	33 (2.8)
4.1.2. American vaccines are effective	206 (17.7)
4.1.3. British vaccines are effective	69 (5.9)
4.1.4. Russian vaccines are effective	14 (1.2)
4.1.5. Indian vaccines are effective	15 (1.3)
4.2. Vaccines are NOT effective at preventing COVID-19 for individuals	11 (.9)
4.2.1. Chinese vaccines are NOT effective	7 (.6)
4.2.2. American vaccines are NOT effective	6 (.5)
4.2.3. British vaccines are NOT effective	0 (0)
4.2.4. Russian vaccines are NOT effective	2 (.2)
4.2.5. Indian vaccines are NOT effective	0 (0)
4.3. The benefits of vaccination to society	20 (1.7)
4.4. Vaccines are NOT effective at preventing COVID-19 in society	1 (.1)
Sub-themes of barriers	
5.1. Belief barriers	17 (1.5)
5.2. Harm barriers	62 (5.3)
5.3. Access barriers	184 (15.8)
Sub-themes of cues to action	
6.1. Government’s recommendation	14 (1.2)
6.2. Experts’ recommendations	25 (2.2)
6.3. Testimony of celebrities	101 (8.7)
6.4. Testimony of ordinary people	114 (9.8)
Sentiment regarding COVID-19 vaccines	
7.1 Pro-vaccine	564 (48.5)
7.2 Anti-vaccine	36 (3.1)
7.3 Not mentioned	562 (48.4)

**Table 4:** The frequency of HBM constructs used by three news agencies.

	Susceptibility	Severity	Self-Efficacy	Benefits	Barriers	Cues to Action
AP	59 (42.4)	63 (45.3)	5 (3.6)	51 (36.7)	101 (72.7)	33 (23.7)
AFP	114 (25.4)	81 (18.0)	11 (2.4)	170 (37.9)	285 (63.5)	126 (28.1)
Reuters	152 (26.5)	87 (15.2)	9 (1.6)	138 (24.0)	298 (51.9)	89 (15.5)
Chi-square	16.57 ***	65.49 ***	2.50	25.02 ***	26.31 ***	24.20 ***
df	2	2	2	2	2	2
*p*	0.00	0.00	0.29	0.00	0.00	0.00

Note: Values inside the parenthesis represent the percentage of n. *** *p* < 0.001.

**Table 5:** HBM constructs and Twitter engagement.

	HBM Variable	Engagement Variables	Mean Ranks of the Group with the HBM Variable Present	Mean Ranks of the Group with the HBM Variable Absent	Mann–Whitney U	Z	*p*
AP	Susceptibility	Retweets	62.36	75.63	1909.50	−1.92	0.06
		Likes	63.58	74.74	1981.00	−1.62	0.11
	Severity	Retweets	73.79	66.86	2155.00	−1.01	0.31
		Likes	73.16	67.38	2195.00	−0.84	0.40
	Self-efficacy	Retweets	54.10	70.59	255.50	−0.90	0.37
		Likes	42.40	71.03	197.00	−1.56	0.12
	Benefits	Retweets	75.49	66.82	1964.00	−1.22	0.22
		Likes	78.46	65.10	1812.50	−1.89	0.06
	Barriers	Retweets	72.61	63.07	1655.50	−1.25	0.21
		Likes	73.18	61.54	1597.50	−1.52	0.13
	Cues to action	Retweets	77.71	67.60	1494.50	−1.26	0.21
		Likes	83.39	65.83	1307.00 *	−2.19	0.03
AFP	Susceptibility	Retweets	170.00	243.71	12,825.50 ***	−5.24	0.00
		Likes	182.58	239.44	14,259.00 ***	−4.04	0.00
	Severity	Retweets	160.62	239.17	9689.00 ***	−4.93	0.00
		Likes	164.59	238.30	10,011.00 ***	−4.63	0.00
	Self-efficacy	Retweets	312.14	222.81	1450.50 *	−2.26	0.02
		Likes	306.86	222.94	1508.50 *	−2.12	0.03
	Benefits	Retweets	220.37	227.82	22,928.50	−0.59	0.56
		Likes	223.54	225.89	23,466.50	−0.19	0.85
	Barriers	Retweets	223.86	226.98	23,046.00	−0.25	0.81
		Likes	222.24	229.80	22,582.00	−0.60	0.55
	Cues to action	Retweets	189.98	238.66	15,936.50 ***	−3.57	0.00
		Likes	210.46	230.67	18,517.00	−1.48	0.14
Reuters	Susceptibility	Retweets	310.32	279.28	28,603.50 *	−1.98	0.048
		Likes	326.90	273.31	26,082.50 ***	−3.42	0.00
	Severity	Retweets	317.58	282.13	18,567.50	−1.84	0.07
		Likes	330.87	279.75	17,411.00 **	−2.65	0.008
	Self-efficacy	Retweets	189.94	289.05	1664.50	−1.78	0.08
		Likes	151.61	289.66	1319.50 *	−2.48	0.01
	Benefits	Retweets	311.45	279.92	26,779.50	−1.95	0.05
		Likes	314.42	278.98	26,368.50 *	−2.19	0.03
	Barriers	Retweets	312.28	260.74	33,738.50 ***	−3.72	0.00
		Likes	312.30	260.73	33,734.50 ***	−3.72	0.00
	Cues to action	Retweets	325.48	280.53	18,202.00 *	−2.35	0.02
		Likes	351.85	275.69	15,855.50 ***	−3.98	0.00

* *p* < 0.05, ** *p* < 0.01, *** *p* < 0.001.

**Table 6:** Sub-themes and Twitter engagement.

	HBM Variable	Engagement Variables	Mean Ranks of the Group with the HBM Variable Present	Mean Ranks of the Group with the HBM Variable Absent	Mann–Whitney U	Z	*p*
AP	4.1.1. Chinese vaccines are effective	Retweets	29.83	72.78	223.50 **	−3.09	0.00
		Likes	27.44	72.95	202.00 ***	−3.28	0.00
	4.1.2. American vaccines are effective	Retweets	207.22	229.46	1251.50	−1.96	0.05
		Likes	85.62	65.70	1166.50 *	−2.40	0.02
	5.3. Access barriers	Retweets	61.53	73.31	1619.50	−1.55	0.12
		Likes	55.46	75.67	1383.00 **	−2.66	0.008
AFP	1.1. Susceptibility of the general public	Retweets	163.74	233.20	7247.00 ***	−3.66	0.00
		Likes	165.08	233.02	7318.00 ***	−3.58	0.00
	1.2. Susceptibility of vulnerable people	Retweets	168.32	236.55	9866.00 ***	−4.18	0.00
		Likes	188.80	232.38	11,422.50 **	−2.67	0.00
	2.1. Severity of the general public	Retweets	160.62	239.17	9689.00 ***	−4.93	0.00
		Likes	164.59	238.30	10,011.00 ***	−4.63	0.00
	4.3. The benefits of vaccination to society	Retweets	62.00	226.10	180.00 *	−2.18	0.03
		Likes	226.01	74.50	217.50 *	−2.02	0.04
	5.2. Harm barriers	Retweets	134.03	228.14	18,950.50 **	−2.76	0.006
		Likes	126.23	228.41	1773.50 **	−3.00	0.003
	5.3. Access barriers	Retweets	214.61	226.85	12,247.50	−0.72	0.47
		Likes	190.42	231.17	10,602.50 *	−2.39	0.02
	6.4. Testimony of ordinary people	Retweets	188.77	230.48	9367.50 *	−2.30	0.02
		Likes	208.70	227.47	10,543.50	−1.04	0.30
Reuters	1.1. Susceptibility of the general public	Retweets	336.46	281.68	12,660 *	−2.44	0.02
		Likes	343.40	280.85	12,236.50 **	−2.79	0.005
	1.2. Susceptibility of vulnerable people	Retweets	299.60	284.53	24,679.00	−0.87	0.39
		Likes	324.68	278.39	21,845.50 **	−2.66	0.008
	2.1. Severity of the general public	Retweets	322.23	281.63	17,494.00 *	−2.06	0.04
		Likes	337.69	279.02	16,211.00 **	−2.98	0.003
	4.1. Vaccines are effective at preventing COVID-19 for individuals	Retweets	311.45	279.92	26,779.50	−1.95	0.05
		Likes	314.42	278.98	26,368.50 *	−2.19	0.03
	4.1.2. American vaccines are effective	Retweets	321.83	281.45	18,032.00 *	−2.08	0.04
		Likes	336.10	278.93	16,804.00 **	−2.95	0.003
	4.2. Vaccines are NOT effective at preventing COVID-19 for individuals	Retweets	496.00	284.18	666.00 ***	−3.80	0.00
		Likes	379.17	286.04	1717.50	−1.67	0.10
	4.2.2. American vaccines are NOT effective	Retweets	505.40	285.59	333.00 **	−2.95	0.003
		Likes	428.30	286.26	718.50	−1.91	0.06
	4.3. The benefits of vaccination to society	Retweets	123.94	292.49	1954.00 ***	−4.13	0.00
		Likes	108.62	292.96	1693.50 ***	−4.52	0.00
	5.2. Harm barriers	Retweets	346.70	282.95	8499.50 *	−2.37	0.02
		Likes	310.30	285.75	9991.50	−0.91	0.36
	5.3. Access barriers	Retweets	244.34	294.19	15,811.50 *	−2.45	0.014
		Likes	231.56	296.17	14,827.00 ***	−3.18	0.00
	6.3. Testimony of celebrities	Retweets	375.18	281.11	7013.00 ***	−3.42	0.00
		Likes	396.26	279.57	6191.00 ***	−4.24	0.00

* *p* < 0.05, ** *p* < 0.01, *** *p* < 0.001.

**Table 7:** News agencies’ sentiments regarding COVID-19 vaccines.

Sentiment	AP	AFP	Reuters	Chi-Square	df	*p*	Total Sample
Positive	71 (51.1)	246 (54.8)	247 (43.0)	14.35 ***	2	0.00	564 (48.5)
Negative	2 (1.4)	8 (1.8)	26 (4.5)	7.78 *	2	0.02	36 (3.1)
Neutral	66 (47.5)	195 (43.4)	301 (52.4)	8.24 *	2	0.02	562 (48.4)

* *p* < 0.05, *** *p* < 0.001.

**Table 8:** Tweets’ sentiments regarding COVID-19 vaccines and Twitter engagement by news agencies.

	Engagement Variables	Sentiment	Mean Rank of the Engagement Variable	Kruskal–Wallis H	df	*p*
AP	Retweets	Positive	76.61			
		Negative	93.00			
		Neutral	62.20			
		Overall model		5.04	2	0.08
	Likes	Positive	78.08			
		Negative	86.50			
		Neutral	60.80			
		Overall model		6.64 *	2	0.04
AFP	Retweets	Positive	223.19			
		Negative	299.81			
		Neutral	224.21			
		Overall model		2.72	2	0.26
	Likes	Positive	227.09			
		Negative	254.25			
		Neutral	221.17			
		Overall model		0.64	2	0.73
Reuters	Retweets	Positive	297.77			
		Negative	339.27			
		Neutral	274.60			
		Overall model		5.30	2	0.07
	Likes	Positive	310.50			
		Negative	270.17			
		Neutral	270.12			
		Overall model		8.34 *	2	0.02

* *p* < 0.05.

## Data Availability

The data presented in this study are available on request from the corresponding author.
